# Beneficial Effects of the Ketogenic Diet on Nonalcoholic Fatty Liver Disease (NAFLD/MAFLD)

**DOI:** 10.3390/jcm13164857

**Published:** 2024-08-17

**Authors:** Damian Dyńka, Łukasz Rodzeń, Mateusz Rodzeń, Dorota Łojko, Sebastian Kraszewski, Ali Ibrahim, Maria Hussey, Adam Deptuła, Żaneta Grzywacz, Alexandre Ternianov, David Unwin

**Affiliations:** 1 Rodzen Brothers Foundation, 64-234 Wieleń, Poland; 2 Department of Psychiatry, Poznan University of Medical Science, 60-572 Poznan, Poland; 3 Department of Biomedical Engineering, Faculty of Fundamental Problems of Technology, Wroclaw University of Science and Technology, 50-370 Wroclaw, Poland; 4 Schoen Inpatient Children’s Eating Disorders Service, 147 Chester Rd, Streetly, Sutton Coldfield B74 3NE, UK; 5 Private General Medical Practice Maria Hussey, Ojcowa Wola 5, 14-420 Mlynary, Poland; 6 Faculty of Production Engineering and Logistics, Opole University of Technology, 76 Prószkowska St., 45-758 Opole, Poland; 7 Primary Care Centre Vila Olimpica, Parc Sanitary Pere Virgili, c. Joan Miró 17, 08005 Barcelona, Spain; 8 Faculty of Health Social Care and Medicine, Edge Hill University, Ormskirk L39 4QP, UK

**Keywords:** nonalcoholic fatty liver disease (NAFLD), metabolic (dysfunction)-associated fatty liver disease (MAFLD), liver, ketogenic diet, low carb, insulin resistance, weight loss, microbiome, self-monitoring, treatment

## Abstract

The prevalence of nonalcoholic fatty liver disease (NAFLD) is likely to be approaching 38% of the world’s population. It is predicted to become worse and is the main cause of morbidity and mortality due to hepatic pathologies. It is particularly worrying that NAFLD is increasingly diagnosed in children and is closely related, among other conditions, to insulin resistance and metabolic syndrome. Against this background is the concern that the awareness of patients with NAFLD is low; in one study, almost 96% of adult patients with NAFLD in the USA were not aware of their disease. Thus, studies on the therapeutic tools used to treat NAFLD are extremely important. One promising treatment is a well-formulated ketogenic diet (KD). The aim of this paper is to present a review of the available publications and the current state of knowledge of the effect of the KD on NAFLD. This paper includes characteristics of the key factors (from the point of view of NAFLD regression), on which ketogenic diet exerts its effects, i.e., reduction in insulin resistance and body weight, elimination of fructose and monosaccharides, limitation of the total carbohydrate intake, anti-inflammatory ketosis state, or modulation of gut microbiome and metabolome. In the context of the evidence for the effectiveness of the KD in the regression of NAFLD, this paper also suggests the important role of taking responsibility for one’s own health through increasing self-monitoring and self-education.

## 1. Introduction

A dramatic increase in the incidence of nonalcoholic fatty liver disease (NAFLD) presents a problem for public health worldwide. NAFLD is a worsening pandemic, which today is not only the most frequent liver disease but also the main cause of morbidity and mortality due to hepatic pathologies. It is estimated that it may affect from 32.4% to 38% of the world’s population, with a significantly higher incidence observed in men [1,2]. Once regarded as a domain of alcohol-addicted individuals, fatty liver degeneration is today also a scourge for patients with metabolic diseases [3,4,5,6]. The relationship between NAFLD and metabolic disorders is so significant that an international expert panel (from 135 countries worldwide) has recently suggested an update of the terminology and a change in the nomenclature of that disease to metabolic (dysfunction)-associated fatty liver disease (MAFLD) [7]. It is particularly worrying that NAFLD is increasingly diagnosed even in children and is closely related, among other conditions, to insulin resistance and metabolic syndrome [8]. The incidence of NAFLD and its intensity are significantly higher in individuals with metabolic risk factors and they increase in parallel with increasing grade of obesity and type 2 diabetes mellitus (75% of patients with type 2 diabetes also have NAFLD). It is expected that by 2030 the incidence of NAFLD-induced liver cirrhosis will double (in relation to 2016) [2,9]. Another paper suggests that the incidence of NAFLD alone may triple by 2030 [10]. This constitutes a real challenge for contemporary medicine and for the authorities designing healthcare systems worldwide.

NAFLD is a metabolic disease in which an excessive accumulation of fatty tissue occurs in the liver (the presence of steatosis in >5% hepatocytes), which is associated with insulin resistance. The term NAFLD was introduced in 1986 [11] and the disease includes two main stages, i.e., nonalcoholic fatty liver (NAFL) (in which the inflammatory condition and liver injury are slight) and nonalcoholic steatohepatitis (NASH) (in which a significant inflammatory condition and liver injury are present) [12]. Intracellular accumulation of fatty acids (from hepatic de novo lipogenesis, visceral adipose tissue lipolysis, excessively high energy content in the diet, and other factors) in the course of MAFLD leads to lipotoxicity (which can lead to cell damage, cell death, and damage to cellular organelles). This in turn leads to a chronic inflammatory state (among others, as a result of the influence of toxic lipids on cell damage by modifying the functions of mitochondria or the endoplasmic reticulum, directly influencing the modification of intracellular signaling pathways, or interacting with specific proinflammatory kinases in the cell). Fatty acid accumulation can also more directly enhance the production of reactive oxygen species (oxidative stress). An additional pathway involves mitochondrial dysfunction mediated by β-oxidation and peroxisomes, which activates a hepatic inflammatory response. In this way, and through a number of other mechanisms (e.g., through disruptions in the functioning of the gut microbiome), MAFLD progresses to MASH [13,14], although the exact mechanisms of progression are most likely not yet fully elucidated. Due to the complexity of the pathogenesis and of the clinical course of the disease, MAFLD is characterized by non homogeneity and diversity of clinical phenotypes. Biopsy is the gold standard of MAFLD diagnosis, although the disease is commonly detected, among other methods, by ultrasonography (USG) or computed tomography (CT). Importantly, despite the fact that the disease is so widespread, most patients remain undiagnosed and no MAFLD treatment methods have been approved [15,16,17].

MAFLD is an increasing problem, not only in healthcare, but also in terms of its economic aspects. The costs associated with MAFLD differ depending on the methodology, database, country, and specific phenotype of the disease, so a full estimation of all related costs (including indirect ones) is not possible. However, all sources agree that these costs are huge, compared to individuals without the disease [18]. It is worth noting that some cases of MAFLD progress to MASH, which is another stage of hepatic steatosis that carries a significant risk of cirrhosis. According to Younossi et al., the predicted cost of healthcare in patients with nonalcoholic steatohepatitis (NASH) (currently metabolic dysfunction-associated steatohepatitis (MASH) [19] in the USA alone in the years 2020–2039 (as the total cumulative cost) is as high as USD 1662.36 billion (USD 1208.47 billion in obese patients with MASH and USD 453.88 billion in nonobese individuals with MASH); this corresponds to an increase in the cost per patient from USD 3573 in 2020 to USD 6601 in 2039 [20]. Importantly, MASH is the most rapidly increasing indication for liver transplantation in the United States; therefore, the huge costs associated with the procedure should be taken into account [21]. The estimated cost of a single transplantation can even exceed USD 1 million when peritransplant costs are taken into consideration [22,23]. For that reason, this article should primarily serve as an appeal to the authorities requesting that they take these data into account and update their knowledge of the possibilities of regression of many chronic diseases, exemplified by fatty liver degeneration, as the key method of improving the overburdened and underfinanced healthcare systems worldwide. Concentrating on prophylaxis, education, and innovative therapeutic methods, including the use of artificial intelligence and modern applications, may play a significant role in reducing those enormous costs and, more importantly, in improving patients’ quality of life.

Alarming data suggest that the awareness of patients with MAFLD is low. The results of the study by Alqahtani et al. have demonstrated that almost 96% of adult patients with MAFLD in the USA were not aware of their disease [24]. It turned out that even among patients in whom MAFLD was diagnosed during a check-up, a majority of them (55.3%) were not provided with any education or guidelines concerning the treatment, and only 40.2% of patients diagnosed with MAFLD visited clinical departments [25].

A ketogenic diet (KD) may be a new therapeutic option in the treatment of MAFLD. It has already been used in the treatment of epilepsy (as an official indication) for over 100 years, frequently being the last resort when pharmacotherapy has failed (drug-resistant epilepsy). The ketogenic diet has been used for the treatment of intractable epilepsy since 1921 [26]. The mechanism by which it controls seizures is likely rooted in its ability to induce nutritional ketosis, thereby safely mimicking the fasted metabolic state while avoiding the risks of starvation and malnutrition. This intervention has been demonstrated to effectively control seizures in those with drug-resistant epilepsy. While on a KD, the insulin levels are low enough to allow an increased production of ketone bodies (i.e., β-hydroxybutyrate, acetoacetate, and acetone) to take place, leading to a state of ketosis in which the body utilizes fatty acids instead of glucose as the main source of energy [27]. The ketogenic diet is a high-fat, moderate-protein, and low-carbohydrate diet. The total daily distribution of macronutrients is typically 5–10% carbohydrates (and no more than 50 g), 60 to 80–90% fat (depending on the individuals therapeutic need), and 10–30% protein. These ranges allow a degree of flexibility for individual cases, and when used for different purposes. For example, treatment of intractable epilepsy usually requires a much higher proportion of fat while keeping carbohydrate consumption extremely low. The maximum amount of protein that can safely be consumed without compromising the efficacy of the diet remains somewhat unclear. Whilst these percentages provide a rough guide to formulate individual meal plans, it is worth emphasizing that the percentage of each macronutrient strictly depends on the total energy content of the diet per day, which varies slightly for each person. In contrast, standard dietary approaches typically suggest obtaining up to 35% of energy from fat (including up to 10% from saturated fats), 45–75% from carbohydrates (up to 10% from sugars), and 10–35% from protein. In addition to the percentage of macronutrients, important (and often neglected) is the quality of formulation of the diet. For example, diet soda and pepperoni sausage would constitute a ketogenic diet, but no one could claim this would be healthy. Depending on the purpose of a KD, the proportion of macrocomponents can be modified by increasing the percentage of protein in the diet while decreasing the percentage of fat (e.g., the modified Atkins diet). In view of the nature of the ketogenic diet, considering it as a therapeutic option for MAFLD may be justified, as evidenced, among others, by the mechanisms underlying KD and findings from other research [28,29,30,31,32,33,34,35]. Indeed, the ketogenic diet has the potential to have a beneficial effect on NAFLD, if only through the fact of reducing inflammation (which is a significant advantage), reducing body weight (and it is often used for this purpose), and improving glycemia and insulin sensitivity, as supported by its low-carbohydrate (excluding sources of simple sugars and marginalizing fructose) nature. These, and other mechanisms, will be considered in this manuscript.

This article is an analysis of the literature dealing with the effect of ketogenic diet in MAFLD (including a number of mechanisms of action) and it focuses on the potential role of KD in the regression of the disease. We include recent scientific knowledge, at the same time building a bridge between science and real-life situations, pointing to the role a diet change can play in the struggle against disease, not only in health-related but also economic aspects. The aim of this article is not only to present the current state of knowledge of the effect of KD on MAFLD, but also to highlight the importance of making informed health decisions and to indicate the role of self-care and self-education in the prevention and treatment of MAFLD.

## 2. Potential Therapeutic Mechanisms of Ketogenic Diet in MAFLD

### 2.1. Reduction in Insulin Resistance

It is known that insulin resistance is one of the main factors underlying MAFLD development, which has been confirmed in numerous publications [36,37,38]. Insulin resistance occurs as a result of prolonged, elevated blood glucose levels and fatty liver itself. Insulin resistance impairs glucose disposal and leads to hyperinsulinemia in a vicious cycle. Then, fatty liver degeneration starts to occur through increased de novo synthesis of fatty acids in the liver, inflow of fatty acids from the adipose tissue (absence of lipolysis inhibition), and increased production of proinflammatory adipokines and cytokines in the adipose tissue. All this is actually stimulated by hyperinsulinemia and leads to the progressive deposition of triglycerides (TG) in the liver parenchyma and MAFLD development [38,39,40,41,42].

One of the benefits of the ketogenic diet is the reduction in insulin and glucose concentrations in the blood, thus improving sensitivity of cells to insulin action [43]. A number of publications demonstrate that KD decreases concentrations of insulin, glucose, and glycated hemoglobin (HbA1c) through numerous mechanisms of action [44,45,46]. The first of them is body weight reduction (owing to the nature of the ketogenic diet, achieving a calorie deficit is extremely easy and intuitive, with no need to count calories). This, in turn, is associated with an improvement in insulin sensitivity (including as a result of the mechanism of increasing the level of adiponectin (the concentration of which is lower in obese people and increases during the reduction in fat tissue), which has anti-inflammatory effects and improves insulin sensitivity), reduction in glycaemia and insulin concentration values, and, consequently, with an improvement in MAFLD [44,47,48]. It is one of the key mechanisms, although not the only one, since low-carb diets have been found to improve the sensitivity to insulin, even in the absence of major weight loss [49,50,51,52,53]. The second mechanism of action is associated with the absence of monosaccharides (and significant reduction in the total amount of carbohydrates to <50 g daily) in the ketogenic diet, which directly prevents excessive fluctuations of glucose levels and insulin spikes [34,54,55,56]. The stabilization of glycaemia and insulin concentration contributes to an improvement in the MAFLD condition. The third mechanism consists of the fact that a ketogenic diet almost entirely eliminates fructose (present, among others, in fruit juices, high-fructose syrups, or fruits), which can disturb insulin signaling in the liver (including as a result of mechanisms such as promoting de novo lipogenesis (DNL), inducing endoplasmic reticulum (ER) stress, impairing fatty acid oxidation (FAO), and inducing hepatitis). An elimination of that component would, thus, exert a favorable effect on insulin sensitivity [57]. Detailed information regarding fructose is described in Section 2.3. Another possible mechanism includes the effect of ketone bodies themselves, and of the state of ketosis, on insulin sensitivity and MAFLD [29]. It is also known that an increase in their concentration, as an energy source alternative to glucose, is associated with a reduced utilization of glucose (and, consequently, a lower amount of insulin needed). The state of ketosis can also improve insulin sensitivity due to a reduction in oxidative stress, inflammatory condition, and improvement in mitochondrial efficiency [31,58,59].

### 2.2. Body Weight Reduction

Although also found in individuals with normal body weight, nonalcoholic fatty liver disease develops more frequently in patients with excessive body weight. A recent meta-analysis revealed that the prevalence of MAFLD worldwide in patients with overweight accounts for 69.99% (on average, NAFL (currently MAFL—metabolic associated fatty liver) 42.49% and NASH (MASH) 33.50%), while in obese patients that percentage is 75.27% (on average, NAFL (MAFL) 43.05% and MASH 33.67%) [60]. Other data suggest that in cases of extreme obesity, even 90% of patients can simultaneously have MAFLD [61]. Reduction in body weight by only >5% results in a detectable alleviation of fatty liver degeneration, while ≥10% provides the best effects including, among others, regression of fibrosis and disappearance of MASH [62,63].

A ketogenic diet works well for reduction in body weight [64], and it may be the most effective therapeutic option for many patients. Compared to standard diets, a KD provides a stronger feeling of satiety [65]; therefore, weight loss can be achieved without significant hunger, mood changes, and irritation (which occur when on standard weight-reducing diets) [66,67]. Importantly, the aforementioned mechanisms, together with sensitization to insulin (described in Section 2.1), create a calorie deficit in a natural way that can be achieved easily, without focusing on counting calories. In their systematic review based on data analysis, Gibson et al. demonstrated that the state of ketosis significantly suppresses appetite, while ketogenic diet prevents appetite increase, even in spite of body weight loss (negative calorie balance) [68]. This happens even with a great body weight loss, e.g., reduction by 17% in relation to the initial value, and the sensation of hunger significantly increases only after regression of the state of ketosis [69]. Roekenes and Martins revealed in their publication that all available evidence suggests that KD inhibits the secretion of ghrelin (a “hunger hormone” released in response to body weight loss on standard diets) in spite of body weight loss [70]. It was demonstrated in one study that a 10-week-long ketogenic diet applied in patients with type 2 diabetes mellitus not only led to a significant improvement in glycaemia values and dose reduction or complete withdrawal of antidiabetic drugs in 56.8% of patients, but also to body weight loss (by 7.2% on average). Importantly, despite a negative calorie balance, in the 10th week of the study the patients reported a less intense sensation of hunger than at its beginning [71]. Even if body weight reduction is at a similar level compared to a high-carb diet (a similar calorie deficit), a KD can additionally improve metabolic parameters while being safe to use [47,72,73,74]. 

### 2.3. Elimination of Fructose

In the context of MAFLD, fructose seems to be a particularly undesirable sugar. In view of its nature, it should be treated separately from other monosaccharides, and carbohydrates in general. According to Lustig, it can be described as “alcohol without the buzz” since it is metabolized similarly to ethanol and, thus, to some extent, it can also cause similar toxic health effects [75]. Most people realize that alcohol damages the liver. It is worth noting, however, that consumption of excessive amounts of processed products rich in sugar or HFCS (high-fructose corn syrup) can also have a detrimental effect on the liver. This is also true of excessive consumption of fructose from fruit juices, which are not infrequently used as a substitute for water, for hydration, and often given to children by their parents [76,77,78]. It has been postulated that excessive fructose intake may be a major cause of MAFLD development [79]. The association between consumption of fructose-containing food and increased incidence of MAFLD was confirmed by a meta-analysis in 2023 [80]. Fructose (especially in excess) is both an inducer and a substrate of hepatic de novo lipogenesis, a process that contributes to the development of steatosis in the liver (additionally causing insulin resistance and dyslipidemia). Also, it increases ectopic lipid accumulation in the liver, cellular stress, and liver inflammation, just to list some of the negative mechanisms of fructose action [81]. Fructose also has the potential to contribute to leptin (the satiety hormone) resistance, which can ultimately lead to disorders of the satiety center [82,83]. 

Fructose may be natural (mainly from fruit and fruit juices) and industrial, in the form of HFCS (high-fructose corn syrup) and in the disaccharide sucrose, found in lots of processed foods and sweetened beverages (which are the worst source of fructose). Leaving aside the obvious negative effect of industrial fructose, it turns out that potentially healthy 100% fruit juices may also carry a number of risks. The fructose content of such juices is up to ten times that of whole fruit and can be higher than that of sugar-sweetened beverages (67 g of fructose in 1 liter of 100% apple juice) [79,84,85,86]. Although whole fruits are better than industrial sources of fructose and fruit juices (due to their content of bioactive phytonutrients, such as phytosterols, catechins, proanthocyanidins, or resveratrol), the presence of sugars (mainly fructose) may offset the beneficial properties of these components, tipping the scales against fruit consumption in MAFLD [87]. One randomized controlled study compared the effect of consuming more than four servings of fruit per day vs. fewer than two servings of fruit per day for six months in patients with MAFLD. The group consuming more fruit was characterized, among others, by greater hepatic steatosis, dyslipidemia, worse glycaemia, and elevated levels of enzymes, such as alanine transaminase (ALT), aspartate transaminase (AST), gamma-glutamyl transpeptidase (GGTP), or alkaline phosphatase (ALP) [88]. That study demonstrated that even potentially healthy fruits combined with caloric excess can be detrimental to patients with fatty liver degeneration. With this in mind, excessive fruit intake may not be a good option for patients with MAFLD (who are often also overweight/obese), and they may be added to the diet only after hepatic steatosis is reversed.

Fructose in the ketogenic diet is eliminated or at least limited to marginal levels, a direct result of the low-carb (and sugar-eliminating) nature of this diet. Considering this on the one hand, and the negative effects of fructose on fatty liver degeneration on the other, it can be concluded that individuals on a ketogenic diet are not exposed to one of the main negative factors exacerbating MAFLD, i.e., fructose excess, as it is naturally eliminated from their diet.

### 2.4. Elimination of Monosaccharides

It is known that consumption of monosaccharides, especially in excess, has a proinflammatory effect on the entire body. It is postulated that the presence of sugar and processed food (often rich in monosaccharides) in the diet may be a key factor leading to inflammation and its exacerbation [89,90,91,92]. On the other hand, it is known that MAFLD, and MASH in particular, has a proinflammatory background [12,93]. This is particularly relevant given how significantly a proinflammatory diet contributes to an increased risk of MAFLD, even independent of other factors, as shown in a 2023 publication [94]. Consumption of monosaccharides (due to their simple chemical structure) significantly increases serum glucose and insulin levels, causing negative health effects [54]. Repeated releases of insulin and glucose as a result can contribute to the development of insulin resistance, one of the main negative factors exacerbating fatty liver, as described in Section 2.1. On the other hand, insulin resistance itself exacerbates inflammation, if only by increasing the production of proinflammatory adipokines and cytokines in adipose tissue or the accumulation of proinflammatory macrophages, which creates a kind of a vicious circle [41,95,96].

By eliminating simple sugars, the ketogenic diet has the effect of reducing inflammation, on the one hand, and lowering and stabilizing glucose levels, on the other, reducing the risk of developing insulin resistance (thus, MAFLD). The elimination of one of the key proinflammatory factors, i.e., monosaccharides, is natural in individuals on KD, as this is due to the nature of their diet. KD, thus, shows an advantage over standard diets, which allow up to 5% of energy from monosaccharides [34,97].

### 2.5. Limitation of Carbohydrates

Complex carbohydrates characterized by a low glycemic index (GI) are considered better (than monosaccharides) because of their lower glucose and insulin release, and diets with a low GI and glycemic load (GL) are considered anti-inflammatory. Even so, complex carbohydrates are broken down to glucose in the body anyway, a process that takes longer than the readily available (as an energy source) monosaccharides [54,98]. In both cases, however, there is a significant increase in glucose and insulin levels. The authors of a 2022 publication suggest that complex carbohydrates may have an even greater effect on postprandial insulin surge than sucrose [99]. In addition, a meta-analysis demonstrated that replacing monosaccharides (glucose, fructose) and sucrose with starch had no effect on HbA1c concentration [100]. Another meta-analysis found that each 10% reduction in total carbohydrates has a lowering effect on fasting blood glucose (by an average of 0.34 mmol/L) and HbA1c (by an average of 0.20 HbA1c%). Importantly, even after 12 months, the downward trend in HbA1c was still evident [101]. People with MAFLD are recommended to follow the Mediterranean dietary (MD) model, in which the majority of energy comes from complex carbohydrates [102]. This is the main difference between KD and MD. Common features of both diets include low processing (in line with the original premise), avoiding simple sugars, high vegetable intake (recommended in both MD and KD), and consuming larger amounts of fats such as olive oil, fatty fish, avocados, and nuts (although it should be noted that even more fat is consumed on KD). KD usually allows for the consumption of a larger amount of total meat as well as red meat alone, compared to MD. It has been postulated that the Mediterranean diet (MD) may have a preventive effect on MAFLD, as shown in some studies [103,104,105,106]. On the other hand, the 2023 study compared glucose and insulin surge at nine time points (at baseline and after 10, 20, 30, 45, 60, 90, 120, and 180 min) after a standard Mediterranean diet meal and after a ketogenic diet meal of the same calorie content. It turned out that the glucose and insulin levels as well as insulin secretion rates were many times lower after the ketogenic meal compared to the MD meal. From an initial insulin value of 40 ± 4 pmol/L, there was an increase after eating the Mediterranean diet meal to maximum values of 497 ± 101 pmol/L after 20 min, while the maximum insulin level after the ketogenic meal was only 88 ± 12 pmol/L after 30 min (compared to an initial value of 44 ± 5 pmol/L), a huge difference between the two groups [107]. Continuous glucose monitoring (CGM) devices, which allow for real-time feedback, may be helpful as a motivator for long-term lifestyle changes, as increasingly discussed in publications [108,109,110]. The stability of glycaemia achieved using a low-carb or ketogenic diet can be an extremely important incentive, so that achieving a certain health outcome can be much easier.

In addition to its effect on carbohydrate metabolism, lowering total dietary carbohydrates may have an anti-inflammatory effect. Karimi et al. demonstrated that the total carbohydrate intake is associated with an increased risk of inflammation, while no increased risk of inflammation has been observed that would be associated with total dietary fat [111]. The authors suggest reconsidering global guidelines that rely on high-carbohydrate dietary models. The anti-inflammatory effect of carbohydrate restriction was also confirmed in a randomized controlled study in which the authors concluded that a low-carb diet reduced inflammation, which was not observed in the case of low-fat diet [112]. This is also supported by the results of other studies [113,114,115]. One study, moreover, found that a higher carbohydrate intake was associated with fatty liver degeneration in patients with type 2 diabetes aged ≤ 50 years [116].

In consideration of the foregoing, on the one hand, there is a potential anti-inflammatory effect of total carbohydrate restriction. On the other hand, given the high post-meal glycaemia and insulin values of the Mediterranean diet, the ketogenic diet is significantly better in controlling these parameters, which directly translates into a lower likelihood of occurrence of the main risk factor for MAFLD, i.e., insulin resistance. In view of this, even the most low-glycemic carbohydrate-based diets will still be characterized by a higher GI than the ketogenic diet, where the low GI is due to the nature of the diet and is achieved automatically when using this dietary model. 

### 2.6. Anti-Inflammatory State of Ketosis

The inflammatory background of nonalcoholic fatty liver degeneration, particularly MASH, makes it reasonable to consider the inflammatory process as one of the main factors exacerbating the course of this disease [12,117,118]. It is known now that activation of the NLRP3 inflammasome, which is significantly increased in MAFLD patients, is strongly associated with the development of this disease. That protein complex plays a key role in the inflammatory process and is a kind of command center for proinflammatory cytokines. This means that its activation (as a result of unfavorable factors) is associated with an increase in inflammatory markers and often causes chronic inflammatory condition [34,119,120]. Currently, pharmacological treatment specifically targeting the NLRP3 inflammasome is suggested as one of the treatments for MAFLD, which includes as many as four categories of agents, i.e., NLRP3 inhibitors, inhibitors of inflammasome components, pharmacological chemotherapeutics, and botanical drugs [119]. However, the safety of their use remains questionable.

The ketogenic diet, through the state of ketosis, exerts a natural strong anti-inflammatory effect, which can even be considered as one of the main advantages of this dietary model [121,122,123,124,125]. This effect can be observed by determining C-reactive protein (CRP) levels [126,127]. Alleviation of the inflammatory condition by KD can even be seen when using an even more sensitive indicator of inflammation, hs-CRP (decrease from 3.45 ± 3.69 mg/dL to 1.80 ± 2.32 mg/dL after 30 days), as shown in a 2023 study [128] and reported in other publications [129,130]. KD also shows the ability to reduce proinflammatory interleukins (including IL-1β, IL-2, IL-4, IL-6, IL-18) and tumor necrosis factor alpha (TNFα), it inhibits the activity of cyclo-oxygenase-2 (COX-2) and inducible nitric oxide synthase (iNOS), and reduces the level of inflammation-related transcription nuclear factor kappa B (NFκB) (which plays a key role in the transcription of coding genes inflammation-related proteins) [131,132,133]. It also appears to have the property of reducing (just like fasting) the activation of NLRP3 inflammasome, which means that it works through the same mechanism as some pharmacological drugs, but in a natural way, without negative health consequences. The main ketone body (β-hydroxybutyrate) BHB, produced as a result of the KD, has an inhibitory effect on NLRP3 inflammasome (via multiple mechanisms) [134]. The reliance on ketone bodies is so important that even just administering them in exogenous form reduced the values of the inflammation indicators, i.e., IL-1β, IL-6, IFN-γ, MCP-1, and RANTES [135]. BHB inhibits the NLRP3 inflammasome, preventing K(+) efflux and reducing adaptor-apoptosis-associated-speck-like protein (ASC) oligomerization and formation of specks (which are large ASC protein complexes following inflammasome activation). KD (via BHB) attenuates activation of caspase-1 and secretion of IL-1β in the models of diseases mediated by NLRP3 [133,136]. There are a number of potential mechanisms for KD’s effect on reducing NLRP3 inflammasome activation. The authors of a 2023 publication point to possible effects through, among others, intracellular signaling, reactive oxygen species (ROS), endoplasmic reticulum (ER), stress, autophagy, mitochondrial metabolism, post-translational modifications, and receptor-mediated action [134]. Thus, the multifaceted effect of the ketogenic diet on one of the major negative factors associated with MAFLD (inflammatory condition) makes it another potential therapeutic mechanism of KD in people with nonalcoholic fatty liver degeneration.

Oxidative stress exacerbates inflammation, in addition to its direct impact on MAFLD, leading to advancement of the disease process into its subsequent, more dangerous stages. The literature also suggests that several other mechanisms may be at play, including the effects of oxidative stress on 1. lipid metabolism, 2. damage to intracellular structures including several organelles involved in key metabolic pathways (mitochondrial and endoplasmic reticulum dysfunction are some examples), and 3. directly causing lipotoxic liver damage, which may ultimately contribute to the development and progression of MAFLD [137]. Given the favorable impact that the KD has on these key mechanisms, including reduced inflammation, reduced oxidative stress, and enhanced mitochondrial function, the KD is likely to play a beneficial role in preventing the progression of this disease. The mechanisms underpinning the role of the KD on liver function were described in a review by Paola and Cerullo. The authors discussed a number of factors, including 1. improved mitochondrial function (stimulating mitochondrial formation, mitochondrial dynamics and bioenergetic pathways) and 2. activating several key factors that alleviate oxidative stress, as possible routes through which the KD contributes to reduced inflammation and improved overall liver function [59].

### 2.7. Modulation of Intestinal Microbiome and Metabolome

Research is increasingly focusing on the role of intestinal microbiome and metabolome in the course of MAFLD. It has been found that dysbiosis of gut microbiome is associated with the etiopathogenesis of this disease from the early stages to the development of MASH and liver cirrhosis. The relationship is confirmed by the fact that the use of probiotics, prebiotics, synbiotics, postbiotics, and parabiotics alleviates the dysbiosis and improves liver function parameters [138,139]. Associated with the course of MAFLD is a change in the composition of the gut microbiome, frequently an increase in the abundance of harmful microorganisms at the expense of beneficial ones. The result is the creation of a proinflammatory intestinal environment, disruption of the integrity of the intestinal barrier, and greater exposure of the liver to harmful agents (including dietary ones), which directly exacerbates the course of MAFLD [140]. The authors of the publication explicitly list which bacterial phyla, families, and genera are most often associated with the disease (and to some extent also with its further stage (MASH)). At the phylum level, it is an increase in Proteobacteria, at the family level—an increase in Enterobacteriaceae and a decrease in Rikenellaceae and Ruminococcaceae, and at the genus level, an increase in Escherichia, Bacteroides, Dorea, and Peptoniphilus and a decrease in Faecalibacterium, Coprococcus, Anaerosporobacter, and Eubacterium [141,142,143,144,145,146,147]. There is even a link between liver cirrhosis and the presence of Prevotella, Veillonella, and Streptococcus strains in the oral cavity, which are rare in healthy individuals [148]. One publication has even shown that the signature of intestinal microbiome can predict the development of MAFLD, using obese women as an example [149]. In view of the foregoing, the microbiome may be a kind of biomarker of disease severity, as evidenced by publications suggesting a causal role of the microbiome in the progression of the disease [150]. An important role of the gut–liver axis is postulated, whose dysregulation affects the pathogenesis of MAFLD through disruption of intestinal integrity, endotoxemia, inflammatory condition, changes in bile acids profiles, and bacterial metabolites [151]. The occurrence of this axis and liver function make the liver particularly vulnerable to toxins from intestinal absorption, as it is the first line of defense against bacterial metabolites. Lipopolysaccharide (LPS), as one of the main proinflammatory products of the intestinal microbiota, can induce lipopolysaccharidaemia and, thus, adversely affect MAFLD [152,153]. It is also worth remembering that increased dietary fructose contributes increased intestinal fructose (as it is absorbed much slower than glucose), which leads to bacterial fermentation and the development of pathogenic bacteria, causing dysbiosis [154]. In addition to dysbiosis, it can also disrupt the integrity of the intestinal barrier (loss of tight junction proteins in the small intestine), thus contributing to MAFLD development [155]. A negative effect, also through the gut microbiome, is generally exerted by a high-sugar diet [156]. 

The ketogenic diet shows a strong effect on modulating the intestinal microbiome and metabolome. This mechanism is even being investigated as a major therapeutic effect in epilepsy, as the therapeutic effect of KD shows a correlation with changes in the intestinal microbiome [157,158]. This nutritional model may affect the composition, function, and diversity of the gut microbiome [159,160]. KD-specific modulation of the microbiome is associated with a reduction in intestinal proinflammatory Th17 cells [161]. On the other hand, it has been demonstrated in an animal model that a KD-specific metabolic state can alter the inflammatory response associated with acute endotoxemia. The high-carb group had higher levels of IL-6 and TNF-α, as well as five times higher expression of hepatic NFκB compared to the ketogenic group [162]. Moreover, it appears that a ketogenic diet, contrary to appearances, can increase the amount of all short-chain fatty acids (SCFAs) in the intestine (including the main one, sodium butyrate), as shown in a six-month-long study [163]. At the same time, it is also known that sodium butyrate, by regulating the intestinal microbiota, can alleviate fatty liver degeneration caused by fructose [164]. It has further been shown that patients with end-stage MAFLD, or liver cirrhosis, had lower levels of fecal SCFAs (mainly butyrate and acetate) [165]. Microbial metabolites, SCFAs in particular, are one of the subjects of studies on the course of MAFLD [166], and their blood levels (isobutyrate and methylbutyrate) show a favorable, significant, and negatively correlated relationship with the severity of MAFLD [167].

**Figure 1:**
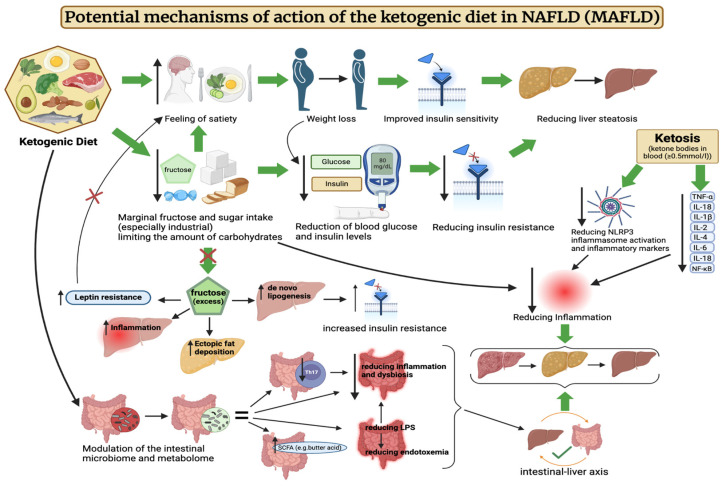
Potential mechanisms of action of the ketogenic diet in NAFLD (MAFLD). The above figure was created with BioRender.com, accessed on 8 August 2024. Agreement number: CR275R2WWY.

Potential mechanisms of action of the ketogenic diet in MAFLD are illustrated in Figure 1.

## 3. Effect of Ketogenic Diet on MAFLD in Humans

**Figure 2:**
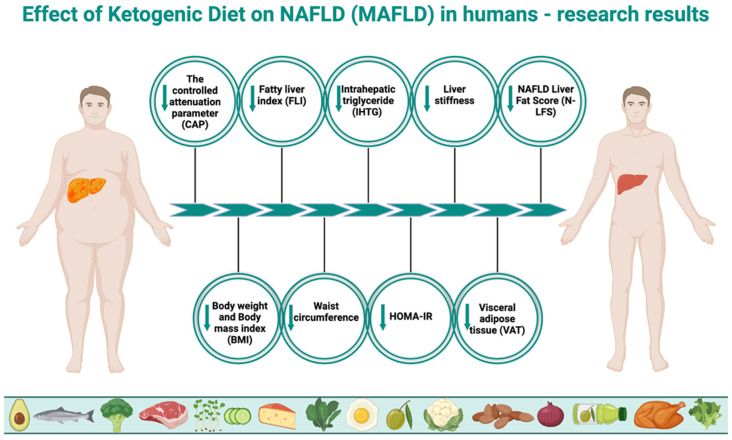
Effect of ketogenic diet on NAFLD (MAFLD) in humans. The above figure was created with BioRender.com, accessed on 8 August 2024. Agreement number: KZ275R2EJK.

The effect of the ketogenic diet on nonalcoholic fatty liver disease is a growing focus of research. Although it is an area that requires more scientific exploration, the results of existing studies are promising. Optimistic conclusions have been drawn by the authors of a 2023 study, which assessed, among other parameters, the efficacy and safety of a very-low-calorie ketogenic diet (VLCKD) for the treatment of MAFLD in 33 patients with excessive body weight (BMI > 25, aged 18–64, who were not taking medications). After 8 weeks on VLCKD, a significant improvement in fatty liver degeneration was observed. The controlled attenuation parameter (CAP) decreased from 266.61 ± 67.96 to 223 ± 64.19, and fatty liver index (FLI) from 62.82 ± 27.46 to 44.09 ± 31.24. In addition, other MAFLD-related indices also changed favorably. Simultaneous reductions were observed, among other parameters, in insulin resistance measured by the homeostatic model assessment for insulin resistance (HOMA-IR) (from 3.11 ± 1.74 to 1.95 ± 0.97), insulin levels (from 12.97 ± 7.19 μU/mL to 8.93 ± 4.29 μU/mL), body mass index (BMI) (from 33.84 ± 6.55 to 30.89 ± 6.38), mass of body fat (MBF) (from 38.47 ± 12.59 kg to 30.98 ± 12.39 kg), waist circumference (from 106.67 ± 15.51 cm to 98.64 ± 16.21 cm), total cholesterol concentration (from 213.49 ± 42.25 mg/dL to 178.09 ± 28.14 mg/dL), LDL (from 140.85 ± 41.07 mg/dL to 113.27 ± 26.94 mg/dL), and triglycerides (from 112.82 ± 58.9 mg/dL to 86.42 ± 42.37 mg/dL). The authors clearly concluded that treatment of MAFLD using VLCKD is both effective and safe [168]. Another, similar study from 2023 also showed the benefits of this dietary model on a larger group of patients (58 women and 29 men, aged 18–64, also not taking medications). The aim of the authors was to evaluate the effect of VLCKD on, among others, white blood cells (WBCs), platelets (PLTs), and hs-CRP concentration (as inflammatory condition parameters) and its simultaneous influence on liver steatosis and liver fibrosis in patients with excessive body mass but with no comorbidities. After eight weeks, a reduction was observed in CAP from 287 (255; 325) to 230 (188; 278), liver stiffness parameters (kPA) from 5.50 (4.30; 6.50) to 5.30 (4.00; 6.50), BMI (kg/m^2^) from 35.59 (6.31) to 32.59 (6.06), waist circumference from 112.15 (16.17) cm to 103.98 (15.71) cm, and mass of body fat (MBF) from 40.17 (13.14) kg to 33.41 (11.91) kg, as well as total cholesterol, LDL, HDL, triglycerides, and hepatic enzymes (ALT, AST, γ-GT). Moreover, there had been a significant reduction in WBC and PLT levels in patients with steatosis (CAP ≥ 215 dB/m). Other parameters, including insulin and glucose concentrations, systolic and diastolic pressures, and the HOMA-IR index, had also been significantly lower after using VLCKD. The authors concluded that VLCKD reduces systemic and liver inflammation, positively contributing to the health of this organ [169]. The authors of a randomized controlled study (the groups consisted of participants who were overweight or obese, grade 1, aged 21–65) compared the effects of a ketogenic diet with placebo (KD + PL) (*n* = 13), a ketogenic diet with a ketone body supplement (LD + KS) (*n* = 12), and a low-fat diet (LFD) (*n* = 12), all with a similar caloric intake. Contrary to frequent allegations that high consumption of saturated fat would increase liver fat, however, the results showed a reduction in liver steatosis. In each group, there was a significant decrease in liver fat without major differences. For KD + PL it was −32%, for KD + KS it was −42%, and for LFD it was −52%. The absolute reduction in liver fat percentage was almost identical, accounting for −2.0% in the KD + PL group, −1.9% in the KD + KS group, and −2.1% in the LFD group. In addition, in the MAFLD subgroup, the ketogenic diet performed better in reducing insulin resistance, as the HOMA-IR index decreased from an average of 4.56 ± 0.90 to 1.48 ± 0.54, while in the LFD group it only decreased from 3.45 ± 1.06 to 2.46 ± 0.64. Similarly, glucose levels decreased from an average of 97.3 ± 6.2 to 84.7 ± 2.81 in the KD group, while they increased from 84.8 ± 6.6 to 86.2 ± 3.34 in the LFD group. The authors pointed out that a six-week hypocaloric diet rich in saturated fat with low carbohydrate intake does not adversely affect liver health, and even slightly improves liver parameters [170]. Results of another randomized controlled study showed that VLCKD (*n* = 20) was more effective than a standard low-calorie (LC) (*n* = 19) diet in reducing body weight, visceral adipose tissue, and liver fat (participants ≥ 18 years of age, BMI > 30 kg/m^2^, recruited among patients referred for weight loss treatment). In the VLCKD group, a greater loss of body weight (−9.59 ± 2.87% vs. −1.87 ± 2.4% in the LC group), visceral adipose tissue (VAT) (−32.0 cm^2^ vs. −12.58 cm^2^ in the LC group), and liver fat (−4.77 vs. 0.79% in the LC group) was observed. The authors suggest that VLCKD may be an effective alternative treatment for MAFLD. However, a limitation of that study is the fact that both groups differed significantly in caloric intake. In the case of VLCKD, it was just 600–800 kcal, while in LC it was 1400–1800 kcal [171]. Interesting results were obtained by Luukkonen et al., who decided to investigate the mechanism underlying the regression of liver steatosis and insulin resistance by a ketogenic diet, despite an increase in circulating nonesterified fatty acids (NEFA), among 10 participants (5 women and 5 men) aged 58.2 ± 2.8 years with a BMI of 31.6 ± 2.0 kg/m^2^. It was found that in spite of a 35% increase in NEFA level, KD reduced intrahepatic triglycerides (IHTGs) concentration by 31%, with a 58% reduction in liver insulin resistance and a 3% reduction in body weight in just six days. The authors indicated that the obtained results could have been affected by an increased net hydrolysis of IHTG (which was attributed to reduced liver insulin resistance) and partitioning of the resulting fatty acids towards ketogenesis (+232%) due to reduced insulin levels (−53%) and hepatic citrate synthase flux (−38%), which is associated with an increased redox status in hepatic mitochondria (+167%) and decreased plasma leptin (−45%) and triiodothyronine (T3) (−21%) concentrations. This study has demonstrated previously undescribed mitochondrial adaptations associated with the reversal of MAFLD through KD, related to the state of ketosis rather than IHTG synthesis. The authors highlight hepatic mitochondrial fluxes and redox state as potential targets for treatment of this disease [31]. Authors of another study set out to assess the effect of VLCKD (45 days) with transition to a hypocaloric low-carb diet (LCD) (for another 45 days) and to identify the predictive factors of MAFLD, among 65 obese patients (BMI > 30 kg/m^2^) aged 18–60, with stable body weight in the last 3 months. After 45 and 90 days, a number of parameters improved, including reductions in body weight, HbA1c, fibroblast growth factor 21 (FGF21), insulin resistance measured by HOMA-IR, hepatic steatosis index (HIS), and triglycerides, as shown in Table 1. HOMA-IR index (positive; R = 0.414) and FGF21 (negative; R = 0.364) have been demonstrated to be independent predictive factors of HSI reduction. The authors suggest a possible role of human FGF21 in alleviating MAFLD as a result of the diets used [172]. The aim of another study was to determine the effect of gender differences on body weight loss and alleviation of MAFLD following VLCKD in obese patients. The study included 42 women and 25 men with severe obesity (BMI ≥ 35 kg/m^2^ and ≥40 kg/m^2^), aged 18–64, with diseases comorbid with obesity (including metabolic disorders, cardiovascular diseases, respiratory diseases, etc.) Body weight loss was greater in men, as was the reduction in γ-glutamyl transferase (γ-GT) levels. The authors concluded that, in this regard, men benefit more from VLCKD compared to women, especially premenopausal women, with the differences disappearing after menopause. Moreover, a greater reduction in HbA1c and HOMA-IR values was observed in men compared with those in women. In view of that, a conclusion can be drawn that the effectiveness of VLCKD in severe obesity is affected by gender differences and, in the case of women, premenstrual status [173]. Interesting results were presented in another publication, the authors of which compared the effects of a one-year-long continuous care intervention (CCI) using a ketogenic diet (262 patients) to a one-year-long usual care (UC) based on the recommendations of the American Diabetes Association (ADA) (87 patients) in patients with type 2 diabetes and their impact on MAFLD and hepatic fibrosis outcomes. Patients were aged 21–65 years, with BMI > 25 kg/m^2^ and diagnosed with type 2 diabetes, excluding patients with significant alcohol consumption, any other cause of liver disease (or secondary causes of MAFLD), and decompensated cirrhosis. Importantly, patients on the ketogenic diet consumed <30 mg of carbohydrate daily, 1.5 g of protein per 1 kg of normal body mass, and were allowed to consume fat until satiety. This was, therefore, not a restrictive diet, as patients chose products according to their own preferences, but their ketosis status was monitored. After one year, patients in the CCI group had a reduction in MAFLD liver fat score (N-LFS) from 3.26 ± 0.21 to 1.30 ± 0.19, while in the UC group there was an increase in N-LFS, from 3.25 ± 0.38 to 3.71 ± 0.35. Body weight loss of ≥5% occurred in 79% of subjects in the CCI group, while only in 19% of subjects in the UC group. MAFLD fibrosis score (NFS) decreased in the CCI group from −0.43 ± 0.08 to −1.14 ± 0.09, and increased in the UC group from −0.62 ± 0.17 to −0.35 ± 0.18. The authors concluded that one year of remotely supported CCI improves MAFLD and liver fibrosis parameters in patients with type 2 diabetes [174]. The most important results in the context of the present study are shown in Table 1. Effects of ketogenic diet on MAFLD in humans are illustrated in Figure 2.

## 4. Role of Education and Self-Treatment in the Process of MAFLD Reversal

### 4.1. A Healthcare System Problem

Healthcare systems are currently failing to manage such the widespread pandemic of MAFLD. Consider its constantly increasing incidence and pessimistic prognosis (even tripling in prevalence by 2030) [1,2,10]. As shown by Lee et al., a majority of patients incidentally diagnosed with MAFLD at their health check-up did not receive any disease management advice, and only 40.2% of those diagnosed visited the clinic [25]. In view of this, the issue is downplayed not only among patients but also among doctors. The issue of physicians’ lack of adequate preparation has been addressed in a number of publications, concluding that physicians underestimate the significance of the problem and lack sufficient knowledge regarding the management and assessment of MAFLD [175,176,177,178,179].

### 4.2. Key Role of Self-Education and Self-Treatment

Given, on the one hand, the above-described inefficiencies in the healthcare system in respect of MAFLD and, on the other hand, the lack of awareness (in up to 95% of individuals) and downplaying the disease by patients [24,25], the role of self-education may be crucial. A study by Stine et al. found that lack of education was one of the key barriers to implementing physical activity in MAFLD patients, which demonstrates how important that factor is [180]. Self-education of patients will reduce complications of the disease and increase the chances of reversing fatty liver degeneration and, on the other hand, educated healthy individuals will reduce the risk of MAFLD development through making informed decisions. Adequate education about MAFLD and other metabolic diseases is an essential tool for taking responsibility for one’s own health and initiating self-treatment, without relying solely on medical care (which may often be insufficient). Self-efficacy has been shown to play a key role in self-treatment, which has shown a significant correlation with self-monitoring outcomes and is the main determinant of MAFLD self-treatment [181]. The significant role of education was confirmed by the study by Vilar-Gomez et al., referred to in Section 3, which demonstrated the superiority of education and remote care (with respect to a ketogenic diet) over standard physician dietary care according to the ADA recommendations in improving liver steatosis in patients with type 2 diabetes within one year [174].

### 4.3. Self-Monitoring and Self-Education Tools

Patients can educate themselves in any way they wish, be it standard education by medical staff, reading books, e-books, guides, searching for information on the internet, or on various web portals, and also using other technologies [182,183]. It is important that patients do not rely on medical care only, but that they are aware of their disease, able to monitor their condition, and conduct the process of MAFLD self-treatment. The possible tools may include the continuous glucose monitoring (CGM) technology, which is the use of glucose sensors that show current blood glucose concentration without the need for constant finger-pricking, making it easier to stabilize glucose and ultimately reduce HbA1c levels [184]. It allows patients to increase their self-awareness and better understand their body response, thus making self-monitoring more effective. This is of great importance because chronically elevated serum glucose levels are associated with MAFLD, creating a vicious circle [185]. Individuals switching from their usual diet to a ketogenic diet usually experience a rapid stabilization of glycaemia, which may pay off in increased motivation and willingness to continue a healthy lifestyle [108,109,110]. The same is true of the promising continuous ketone monitoring (CKM) technology [186], which can be used as a tool to monitor the state of ketosis during a ketogenic diet, thus increasing self-awareness concerning one’s own body’s response. Other methods of self-education and increasing the control over one’s own body may include all kinds of mobile applications of all types. They can help minimize self-identified barriers to lifestyle change, such as lack of education, exercise resources, time, and costs [180,187,188]. One randomized controlled study showed that a mobile application performed better (compared to standard care) in weight loss and improvement in metabolic parameters in patients diagnosed with MAFLD [189]. Another study demonstrated that a six-month-long lifestyle intervention based on a mobile application was feasible and acceptable in patients with MAFLD. A majority of participants confirmed that it was easy to use and motivating for an active lifestyle [190]. There are also applications for strictly ketogenic diets, which have been shown in studies to be effective in reducing body weight, among other things [191,192].

With this in mind, self-education and self-monitoring have an invaluable impact on the prevention and treatment of MAFLD as well as other metabolic diseases. The patient should be aware of his/her disease and lifestyle; only then can the process of prevention or treatment proceed optimally. Relying only on professional care in such a heavily overburdened medical system, without taking responsibility for one’s own health and without changing lifestyle, is likely to be ineffective and may even be irresponsible.

## 5. Potential Side Effects of Using a Ketogenic Diet

It is worth clarifying at this point whether any side effects from the ketogenic diet are possible. The authors of one publication decided to investigate this. It was shown that, especially in the first few days after starting, symptoms such as nausea, polyurea, dizziness, halitosis, sluggishness, constipation, palpitations, and muscle pain can occur. Significantly, the same participants were overwhelmingly satisfied with the effects of the ketogenic diet, with the authors concluding by noting that KD gave a very positive overall experience [193]. However, these symptoms are the body’s physiological response to the change in fuel source from glucose to ketone bodies and are referred to as “keto flu” and subside within the first few days/weeks. Other authors point to similar symptoms reported in the early stages of KD use and these included headache, lethargy, fatigue, nausea, dizziness, decreased energy, gastrointestinal discomfort, changes in heartbeat, and feeling faint [194]. It is worth noting that these are symptoms reported by participants (on online forums) using the ketogenic diet alone. This means that they most likely did not carry it out properly, which could have been avoided. Therefore, these cannot be called side effects of the ketogenic diet, but symptoms of a poorly conducted ketogenic diet (and even in this case they are not serious and go away on their own).

## 6. Perspectives

The increasing dissemination of knowledge related to MAFLD offers hope for better management of this disease, both among healthcare professionals and patients (through self-education and self-monitoring). This could lead to a reduction in the number of new MAFLD cases and improved treatment of those currently affected. In the context of KD’s impact on MAFLD, there are several key areas that require further research. Primarily, studies should be long-term and focus on comparing KD with other diets of similar caloric content (or at least ad libitum), particularly examining normocaloric approaches (which would eliminate the weight loss factor). Efforts should also be concentrated on investigating the metabolic pathways through which KD affects MAFLD, as well as exploring KD’s impact on the microbiome in the context of MAFLD development, treatment, and prevention.

## 7. Summary

The ketogenic diet, by its very nature, may be one of the more effective known therapeutic options for nonalcoholic fatty liver disease. This is because it affects a number of key factors from the perspective of MAFLD regression.

One of the main recommendations in MAFLD therapy is to achieve a calorie deficit in order to reduce body weight. It has been found that KD can reduce calorie intake extremely easily without having to track the calories, by maintaining a high level of satiety and, thus, reducing the desire to snack. KD also impacts another factor, crucial from the point of view of MAFLD regression, i.e., insulin resistance. On the one hand, this is caused by monosaccharides and total carbohydrates intake reduction, which results in lower glucose and insulin spikes (and lower HbA1c levels) and, on the other hand, body weight loss itself (achievable easily on KD) reduces insulin resistance. Limiting the supply of those compounds (especially monosaccharides) also reduces inflammation, which is closely associated with MAFLD (particularly with MASH). Inflammation is also reduced by the state of ketosis itself, which is associated, among others, with the inhibition of NLRP3 inflammasome activation. It has been found that β-hydroxybutyrate (the main ketone body) acts by a similar mechanism as some medications, but without any adverse effects, thus providing a double benefit. The problem of another MAFLD-exacerbating factor, i.e., fructose excess, is absent while on the ketogenic diet. This is due to the marginalization or outright elimination of all forms of mono-, di-, and polysaccharides, including fructose. In view of this, any negative consequences for the liver resulting from the consumption of fructose (even more so, the industrial fructose) simply do not occur in KD users. Furthermore, KD specifically improves the intestinal microbiome and metabolome, disorders of which are correlated with MAFLD. 

The available studies in humans have shown beneficial effects of KD on MAFLD regression; however, these are mostly low-calorie diets, which gives an argument to opponents of KD that the observed effects are solely due to weight loss. In view of the foregoing, constructing well-designed studies should be one of the main research aims regarding MAFLD in the coming years. Gender-associated impact of KD on MAFLD is also worth analyzing. In the face of inefficiencies in the healthcare system, patients (in parallel with medical care) should take responsibility for their health and not rely solely on medical care. The role of self-education and self-management is underestimated, and they might relieve the burden on the current system, while increasing patient control and management of their health. It is also worth placing more emphasis on training doctors how to treat MAFLD properly.

In conclusion, it can be said that a ketogenic diet may be an effective tool for MAFLD regression (particularly promising when combined with self-education and self-monitoring). However, there is a lack of well-designed studies in this area; therefore, exploration of this scientific field should be a focus of research in the coming years. Perhaps this could lead to the introduction of KD in the treatment of MAFLD on a broader scale, resulting in improved health of millions of people and significant savings for healthcare systems worldwide.

## Figures and Tables

**Table 1:** Effects of ketogenic diet on MAFLD parameters in humans.

Year of Study, References	Study Aim	Groups, Duration, and Comorbidities Reported with MAFLD	Most Important Results
2023 [168]	Assessment of the effect of a very-low-calorie ketogenic diet (VLCKD) on MAFLD (analyzed with transient elastography (Fibroscan) and FLI) and on other metabolic parameters (including insulin resistance, lipid profile, thyroid function parameters, uric acid, vitamin D) and body composition and anthropometric parameters (BMI, fat body weight and lean body mass, waist circumference) in patients with excessive body mass without apparent comorbidities.	1 VLCKD group (carbohydrates: 20–50 g, protein: 1–1.4 g/kg of ideal body weight, fat: 15–30 g); (33 participants); 8 weeks. Co-occurrence: Obesity and overweight.	Changes in the parameters, expressed as mean values: - Reduction of controlled attenuation parameter (CAP) from 266.61 ± 67.96 to 223 ± 64.19. - Reduction in fatty liver index (FLI) from 62.82 ± 27.46 to 44.09 ± 31.24. - Reduction in HOMA index from 3.11 ± 1.74 to 1.95 ± 0.97. - Reduction in insulin concentration from 12.97 ± 7.19 µU/mL to 8.93 ± 4.29 µU/mL. - Reduction in body mass index (BMI) from 33.84 ± 6.55 (kg/m^2^) to 30.89 (kg/m^2^). - Reduction in fat body mass from 38.47 ± 12.59 kg to 30.98 ± 12.39 kg. - Reduction in ALT concentration from 29.3 ± 24.28 U/L to 25.97 ± 28.45 U/L. - Reduction in γ-GT concentration from 20.18 ± 10.83 U/L to 16.33 ± 8.67 U/L. - Reduction in waist circumference from 106.67 ± 15.51 cm to 98.64 ± 16.21 cm). - Reduction in total cholesterol concentration from 213.49 ± 42.25 mg/dL to 178.09 ± 28.14 mg/dL. - Reduction in LDL cholesterol concentration from 140.85 ± 41.07 mg/dL to 113.27 ± 26.94 mg/dL. - Reduction in triglyceride concentration from 112.82 ± 58.9 mg/dL to 86.42 ± 42.37 mg/dL.
2023 [169]	Assessment of the effect of a very-low-calorie ketogenic diet (VLCKD) on white blood cell (WBC) count and platelet (PLT) count, hs-CRP and changes in liver steatosis and fibrosis in patients with excessive body weight without any apparent comorbidities.	1 VLCKD group (carbohydrates: 20–50 g, protein: 1–1.4 g/kg of ideal body weight, fat: 15–30 g); (87 participants); 8 weeks. Co-occurrence: Obesity and overweight.	Changes in the parameters, expressed as mean values: - Reduction in CAP from 287 (255; 325) to 230 (188; 278). - Reduction in liver stiffness value (kilopascals (kPA)) from 5.50 (4.30; 6.50) to 5.30 (4.00; 6.50). - Reduction in HOMA index from 4.10 (3.04) to 2.28 (1.47). - Reduction in insulin concentration from 16.94 (10.78) μU/mL to 10.25 (6.12) μU/mL. - Reduction in BMI from 35.59 (6.31) (kg/m^2^) to 32.59 (6.06) (kg/m^2^). - Reduction in waist circumference from 112.15 (16.17) cm to 103.98 (15.71) cm. - Reduction in fat body mass from 40.17 (13.14) kg to 33.41 (11.91) kg. - Reduction in white blood cell (WBC) count from 6.72 (1.37) (103/μL) to 5.98 (1.36) (103/μL). - Reduction in platelet (PLT) count from 269.72 (54.25) (103/μL) to 244.91 (58.32) (103/μL). - Reduction in ALT concentration from 31.07 (23.71) (U/L) to 25.80 (21.07) (U/L). - Reduction in γ-GT concentration from 25.31 (16.16) (U/L) to 16.84 (8.69) (U/L). - Reduction in total cholesterol concentration from 196.34 (49.57) mg/dL do 168.80 (40.40) mg/dL. - Reduction in LDL cholesterol concentration from 134.24 (35.51) mg/dL to 113.73 (28.47) mg/dL. - Reduction in HDL cholesterol concentration from 52.49 (13.82) mg/dL to 46.77 (11.94) mg/dL. - Reduction in triglyceride concentration from 112.10 (54.79) mg/dL to 89.34 (37.54) mg/dL.
2021 [170]	Assessment of the effect of a hypocaloric ketogenic diet with or without ketone body supplementation, compared to a hypocaloric low-fat diet on the changes in liver fat content over 6 weeks.	3 hypocaloric groups: 1. Ketogenic diet with placebo (KD + PL) (carbohydrates: 38 g, protein: 100 g, fat: 131 g) (13 participants). 2. Ketogenic diet with ketone supplements (KD + KS) (carbohydrates: 40 g, protein: 99 g, fat: 143 g) (12 participants). 3. Low-fat diet (LFD) (carbohydrates: 259 g, protein: 100 g, fat: 51 g) (12 participants); 6 weeks. Co-occurrence: Overweight	Reduction in liver fat level, on average by: - (−32%) in the KD + PL group. - (−42%) in the KD + KS group. - (−52%) in the LFD group. In absolute values, on average by: - (−2.0%) in the KD + PL group. - (−1.9%) in the KD + KS group. - (−2.1%) in the LFD group. Additionally: - Body weight reduction on average by 8.5% in the KD + PL group, by 8.1% in the KD + KS group and by 6.7% in the LFD group. In the MNAFLD subgroup: - Reduction in HOMA-index in the KD group from 4.56 ± 0.90 on average to 1.48 ± 0.54, and in the LFD group from 3.45 ± 1.06 to 2.46 ± 0.64. - Reduction in glucose concentration in the KD group from 97.3 ± 6.2 on average to 84.7 ± 2.81, and an increase from 84.8 ± 6.6 on average to 86.2 ± 3.34 in the LFD group.
2020 [171]	Assessment of the effect of a weight loss programme based on a very-low-calorie ketogenic diet (VLCKD) on the visceral adipose tissue and liver fat, compared to a standard low-calorie (LC) diet.	2 groups: 1. Very-low-calorie ketogenic diet (VLCKD) (carbohydrates < 50 g, protein: 0.8–1.2 g/kg ideal body weight, fat: 10 g (olive oil)) (20 patients). 2. Standard low-calorie (LC) diet (carbohydrates: 45–55%, protein: 15–25%, fat: 25–35%) (19 patients); 2 months. Co-occurrence: Obesity.	Changes in the parameters, expressed as mean values: - Reduction in visceral adipose tissue (−32.0 cm^2^ in the VLCKD group vs. −12.58 cm^2^ in the LC group). - Reduction in liver fat (−4.77% in the VLCKD group vs. 0.79% in the LC group). - Body weight reduction (−9.59 ± 2.87% in the VLCKD group vs. −1.87 ± 2.4% in the LC group). Significant (*p* < 0.05) changes in biochemical parameters: - Reduction in AST level from 22.88 (8.54) to 18.93 (3.92) in the VLCKD group. - Reduction in HbA1c level from 5.52 (0.42) to 5.41 (0.36) in the VLCKD group. - Reduction in uric acid level from 5.38 (1.19) to 5.10 (1.04) in the VLCKD group. - Reduction in total cholesterol level from 187.18 (43.97) to 173.60 (52.14) in the VLCKD group, and from 184.88 (27.39) mg/dL to 171.00 (24.94) mg/dL in the LC group.
2020 [31]	Assessment of the effect of a short-term ketogenic diet on liver steatosis in participants with excessive body weight, and investigation of the mechanisms underlying the changes observed.	1 group, KD (carbohydrates 6%, protein: 28%, fat: 64%) (10 patients); 6 days. Co-occurrence: Obesity and overweight.	Changes in the parameters. expressed as mean values: - Reduction in IHTG by 31% (from 10.3 ± 2.3% to 7.1 ± 2.0%). - Reduction in HOMA index by 57% (from 3.0 ± 0.5 to 1.3 ± 0.2). - Reduction in body weight by 3% (from 93.5 ± 5.3 kg to 90.7 ± 5.2 kg). - Increase in NEFA concentration by 35% (from 0.55 ± 0.02 mmol/L to 0.74 ± 0.02 mmol/L) - Reduction in fasting glucose concentration by 13% (from 112 ± 3 mg/dL to 98 ± 3 mg/dL). - Reduction in the rates of VCS by 38% (from 188 ± 20 μmol/min to 116 ± 8 μmol/min). - Reduction in plasma leptin concentration by 45% (from 46.5 ± 16.7 ng/mL to 25.6 ± 9.5 ng/mL). - Reduction in plasma T3 concentration by 21% (from 0.85 ± 0.08 ng/mL to 0.67 ± 0.03 ng/mL). - Reduction in insulin concentration by 53% (from 10.9 ± 1.8 mU/L to 5.1 ± 0.8 mU/L).
2020 [172]	To identify predictors of MAFLD improvement as reflected by the reduction in the hepatic steatosis index (HSI), a noninvasive screening tool, in obese patients undergoing a weight loss program.	1 group (65 patients); 45 days on VLCKD (carbohydrates 14%, protein: 46%, fat: 40%) and 45 days on LCD (carbohydrates up to 120 g, protein: 1–1.5 g/kg ideal body weight, fat: rest (up to mean 1150 kcal)). Co-occurrence: Obesity.	Changes in the parameters. expressed as mean values: - Reduction in HSI from 47.5 ± 7.5 to 43.3 ± 6.3 (on day 45) and to 33.5 ± 4.6 (on day 90). - Reduction in FGF21 from 180.1 ± 88.9 ng/mL to 128.7 ± 87.7 ng/mL (on day 45) and to 73.5 ± 55.5 (on day 90). - Reduction in HOMA-IR from 4.5 ± 1.8 ng/mL to 2.2 ± 1.4 ng/mL (on day 45) and to 1.5 ± 0.8 ng/mL (on day 90). - Reduction in insulin concentration from 16.3 (7.8) µIU/mL to 8.1 (8.1) µIU/mL (on day 45) and to 6.4 ± 3 µIU/mL (on day 90). - Reduction in HbA1c from 5.6 ± 0.4(%) to 5.3 (0.4)(%) (on day 45) and a slight increase to 5.4 ± 0.27(%) (on day 90). - Reduction in triglyceride concentration from 125.0 (55) mg/mL to 91 (33) mg/dL (on day 45) and to 90 ± 27.7 mg/dL (on day 90). - Reduction in LDL-C concentration from 127.9 ± 35.3 mg/dL to 103.0 ± 32.2 mg/dL (on day 45) and increase to 120.1 ± 29.5 mg/dL (on day 90). - Reduction in total cholesterol concentration from 208.1 ± 42.0 mg/dL to 171.8 ± 38.2 mg/dL (on day 45) and increase to 193.7 ± 32.3 mg/dL (on day 90). - Reduction in ALT concentration from 22 (13) U/L to 20 (9) U/L (on day 45) and to 16 (9) U/L (on day 90). - Reduction in body weight from 104.6 ± 15.3 kg to 95.1 ± 14.1 kg (on day 45) and to 87.5 ± 12 kg (on day 90). - Reduction in fat body weight from 39,824 ± 10,492 g to 34,078 ± 10,230 g (on day 45) and to 30,064 ± 8923 g (on day 90). - Reduction in visceral adipose tissue from 862.8 ± 295.9 g to 781.6 ± 267.5 g (on day 45) and to 689.81 ± 206.9 g (on day 90).
2020 [173]	To investigate the effects of gender differences on weight loss and MAFLD improvement in patients with severe obesity using a VLCKD.	2 VLCKD groups (carbohydrates: <50 g, protein: 1.4 g/kg ideal body weight, fat: <30 g) divided according to gender: group 1–42 women; group 2–28 men; 25 days. Co-occurrence: Obesity	Changes in the parameters. expressed as mean values: - Reduction in body weight from 137 ± 18 kg to 124 ± 20 kg (in males) vs. from 119 ± 20 kg to 109 ± 20 kg (in females). - Reduction in γGT concentration from 65 ± 42(IU/L) to 40 ± 28 (IU/L) (in males) vs. from 32 ± 30 (IU/L) to 20 ± 13 (IU/L) (in females). - Reduction in HbA1c concentration from 6.5 ± 1.4(%) to 5.9 ± 1.3(%) (in males) vs. from 7.1 ± 5.3(%) to 5.7 ± 0.7(%) (in females). - Reduction in HOMA-IR from 11.3 ± 9.2 to 4.5 ± 4.1 (in males) vs. from 5.5 ± 6.0 to 2.9 ± 2.8 (in females). - Reduction in the amount of fatty tissue from 44 ± 9% to 40 ± 7% (in males) vs. from 50 ± 5% to 47 ± 5% (in females). - Reduction in liver steatosis (grade 3) from 58.3% to 18.8% (in males) vs. from 41.7% to 18.2% (in females) and an increase in the percentage of individuals without steatosis from 0% to 6.3% (in males) and from 5.6% to 15.2% (in females).
2019 [174]	Assessment of the effect of CCI through nutritional ketosis on the surrogate outcomes of MAFLD and liver fibrosis in patients with type 2 diabetes (compared to UC).	2 groups group 1 CCI (ketogenic diet) (carbohydrates: <30 g, protein: 1.5 g/kg ideal body weight, fat: ad libitum) (262 patients) group 2 UC (diet based on the ADA recommendations) (87 patients); 1 year. Co-occurrence: Type 2 diabetes.	Changes in the parameters. expressed as mean values: - Reduction in N-LFS from 3.26 ± 0.21 to 1.30 ± 0.19 in the CCI group and increase in N-LFS from 3.25 ± 0.38 to 3.71 ± 0.35 in the UC group. - Reduction in NFS from −0.43 ± 0.08 to −1.14 ± 0.09 in the CCI group and increase in NFS from −0.62 ± 0.17 to −0.35 ± 0.18 in the UC group. - Reduction in body weight ≥5% in 79% of the patients in the CCI group and in 19% of the patients in the UC group. The following observations, among others inter alia, were made in the subgroup with abnormal ALT activity (at the beginning of the study): - Reduction in HbA1c from 7.50 ± 0.10 (%) to 6.16 ± 0.10 (%) in the CCI group and increase in HbA1c from 7.10 ± 0.21 (%) to 7.32 ± 0.18 (%) in the UC group. - Reduction in fasting insulin concentration from 30.16 ± 1.75 m/UL to 18.01 ± 1.56 m/UL in the CCI group and from 32.15 ± 3.63 m/UL to 30.01 ± 3.41 m/UL in the UC group. - Reduction in HOMA-IR from 9.57 ± 0.60 to 5.18 ± 0.70 in the CCI group and increase in HOMA-IR from 11.51 ± 1.18 to 13.73 ± 1.43 in the UC group. - Reduction in ALT concentration from 37.00 ± 1.24 U/L to 23.55 ± 1.32 U/L (compared to an increase from 37.86 ± 2.56 U/L to 38.04 ± 2.68 U/L in the UC group). - Reduction in AST concentration from 27.11 ± 0.97 U/L to 19.77 ± 0.83 U/L in the CCI group (compared to an increase from 27.69 ± 2.03 U/L to 28.55 ± 1.73 U/L in the UC group). - Reduction in CRP concentration from 6.85 ± 0.50 mg/dL to 4.51 ± 0.50 mg/dL in the CCI group and increase in CRP concentration from 9.41 ± 1.03 mg/dL to 9.84 ± 1.04 mg/dL in the UC group.

## Data Availability

Not applicable.
